# Study on Compressibility According to Mixing Ratio and Milling Time of Fe-6.5wt.%Si

**DOI:** 10.3390/ma17081723

**Published:** 2024-04-09

**Authors:** Jaemin Kim, Seonbong Lee

**Affiliations:** 1 Department of Mechanical Engineering, Keimyung University, Daegu 42601, Republic of Korea; 1114148@stu.kmu.ac.kr; 2 Department of Automotive Engineering, Keimyung University, Daegu 42601, Republic of Korea

**Keywords:** SMCs (soft magnetic composites), MPFEM (multi-particle finite element method), powder metallurgy

## Abstract

Recently, researchers have focused on improving motor performance and efficiency. To manufacture motors with performance and efficiency higher than those of motors manufactured through the additive process, compressibility verification through the parameter control of soft magnetic composites (SMCs) is essential. To this end, this study aims to select suitable powders for manufacturing high-performance, high-efficiency motors by exploring powder mixing ratios and milling times. Through physical property tests, the optimal mixing ratio is predicted using the Multi-Particle Finite Element Method (MPFEM) and powder compression molding analysis, and compressibility is predicted in view of the influence of a change in particle size as a function of the powder mixing ratio and milling time. In addition, based on the result of a comparative analysis of density through experiments and an analysis of internal defects through SEM, a 50:50 mixing ratio of hybrid atomizing and gas atomizing powders milled for 3 h provided the best compressibility. Therefore, the use of SMC cores fabricated using powder compression molding is expected to improve motor performance and efficiency.

## 1. Introduction

In recent years, the importance of soft magnetic composites (SMCs) in motors has increased considerably [1,2]. These composites provide innovative performance and mechanical advantages over traditional steel plates, which makes them essential for improving motor performance. In terms of performance, first, SMCs possess strong magnetic properties and offer more complex magnetic paths and efficient magnetic flows than those of traditional laminated electrical steel sheets [3,4]. Second, SMCs improve the energy efficiency of the entire system by significantly reducing eddy current loss. This is especially important for improving motor performance at high frequencies [5]. Third, the flexible design possibilities of SMCs allow for the creation of freer shapes and structures than those possible with the existing electrical steel sheets. This facilitates new creative approaches to motor design. Finally, SMCs provide economic benefits such as improved energy efficiency, reduced device size, and reduced operating costs. Additionally, mechanically, when a steel plate is punched into the shape of a stator that matches the motor structure, scrap is generated. This process increases cost and energy consumption, but these problems are eliminated when using SMC materials in conjunction with sintering.

In powder compression molding, metal products are manufactured through compression molding using metal powder and, subsequently, sintered below the melting temperature of the metal. The associated process conditions vary depending on product shape and characteristics of the constituent material. A typical powder metallurgy process comprises powder manufacturing, mixing, molding, sintering, and post-processing. In each step, molding temperature, molding speed, sintering temperature, molding pressure, and powder loading are selected as the key factors, and their values are influenced by the product’s fillet dimensions, length-to-outer-diameter ratio, density, and material properties.

In general, microstructure is an important determinant of the performance of SMCs. Microstructure-related parameters include particle size and distribution, milling time, particle shape, porosity, coating thickness, and the presence or absence of a coating [6,7].

The large cross-sectional areas of large particles allow for stronger eddy current circulation at low frequencies, and the resulting attenuated demagnetization field facilitates uniform magnetic domain alignment. These phenomena reduce hysteresis losses, which is advantageous at lower frequencies, where rapid magnetization changes are less important. Conversely, for small particles, hysteresis loss increases at high frequencies because their small cross-sectional area limits current flow. However, this small cross-sectional area is advantageous at high frequencies because it leads to faster magnetic saturation and reduced eddy current loss [8,9,10,11,12,13,14].

In terms of the effect of porosity on magnetic properties, as porosity increases, the effective path length of magnetic flux increases. Moreover, permeability decreases because porosity reduces the overall magnetic response. Hysteresis and eddy current losses increase because the uniformity of the magnetic field inside SMCs is disturbed. In addition, in terms of mechanical properties, pores facilitate cracking in molded products, and as porosity increases, density decreases, which affects structural integrity. Additionally, porosity induces non-uniform stress distribution in SMCs, thereby decreasing the load-bearing area. This increases iron loss due to plastic deformation during compression molding [15,16,17,18,19,20].

Moreover, in powder metallurgy, compressibility is directly related to the manufacturing process of SMCs. In the compression step, as the compression pressure increases, the density of the molded body increases. As the volume fraction of the magnetic material in an SMC increases, the permeability of the SMC increases at relatively low frequencies and converges when the compression pressure exceeds a certain threshold. At relatively high frequencies, the permeability decreases because voids are removed, and strong eddy currents are generated owing to deterioration of the surface insulating layer [21,22,23].

Annealing is performed to reduce the hysteresis and eddy current loss caused by residual stress in SMCs, which is generated during powder manufacturing and compression. Annealing affects nanocrystallization, refines the domain structure, induces anisotropy, and causes microstructural changes. Products annealed at high temperatures and relatively low frequencies exhibit smaller magnetic loss than those annealed at low temperatures. However, at relatively high frequencies, products annealed at low temperatures exhibit smaller magnetic loss than those annealed at high temperatures [24,25].

However, there is insufficient research on the use of Fe-6.5wt.%Si powder in SMCs, especially those used as potential replacements for stators produced using conventional sheet-stacking methods. In this study, we explore the milling time and mixing ratio of Fe-6.5wt.%Si powder for fabricating an SMC core, and the three main study objectives are as follows:

(1) Derive the optimal mixing ratio based on the experimental results.

By molding a toroidal core from powders prepared with various mixing ratios, the optimal mixing ratio is determined experimentally.

(2) Conduct a Multi-Particle Finite Element Method (MPFEM) compressibility study—milling time and particle size.

Based on the physical property test results, MPFEM is used to study compressibility as a function of milling time and particle size.

(3) Verification through comparison of powder compression molding analysis and experiment.

The results of the powder compression molding analysis and experiments are compared to verify the results of this study.

## 2. Materials and Methods

### 2.1. Compressibility According to Powder Characteristics

In previous studies, when only gas atomizing was used, compressibility was not good [26]. Therefore, hybrid atomizing powder and gas atomizing powder were used to evaluate compressibility in terms of the mixing ratio and shape of Fe-6.5wt.%Si, and the ratios were set to 100:0, 70:30, 50:50, and 30:70. Additionally, for high-temperature molding, MoS_2_ and H_3_PO_4_ were used as a lubricant and a binder, respectively. This compressibility evaluation was carried out through toroidal molding experiments, which enable the evaluation of various important properties and behaviors of powder materials under conditions very similar to actual manufacturing processes, and the external shape and density of the resulting products were measured and analyzed.

Thermal diffusivity and thermal conductivity are characteristics that indicate how well a material can transfer heat. High thermal diffusivity and thermal conduction lead to more uniform heat transfer throughout the material, resulting in faster thermal diffusion and thermal conduction. These attributes allow for more uniform densification and shorten the sintering time.

The thermal expansion coefficient affects thermal stress during high-temperature compression molding. The higher the thermal expansion coefficient, the greater the expansion and contraction of the powder, which increases internal stress. This stress can cause warping and cracking of parts, thereby affecting their compressibility.

### 2.2. MPFEM

To evaluate compressibility as a function of powder particle size, milling was performed for 1 h (hour), 3 h, and 5 h to alter the particle size of the spherical gas atomizing powder particles, and an analysis was performed considering the corresponding particle size. Generally, during the compression of free powder metal, the powder is assumed to follow the continuum model, as in the existing Finite Element Method (FEM). However, in the continuum model, the material is assumed to be homogeneous, and there are no individual particles. Therefore, it is difficult to determine the particle behavior of the powder, degree of freedom of rotation, and mutual contact relationship between powders [27,28]. To compensate for these shortcomings, Cundall analyzed inter-particle behavior by using the Discrete Element Method (DEM). However, the DEM often ignores particle behavior, including molecular rearrangement, affine motion, particle rotation, and large deformations [29,30]. Additionally, DEM analysis is conducted at relatively low relative densities. Therefore, it may not be possible to ascertain the exact deformation behavior of a material at the particle contact level. By contrast, the MPFEM, developed by Procopio, compensates for these shortcomings [31,32]. In this simulation, the defined motions of the lateral and axial rigid body boundary velocities are changed symmetrically to maintain the specified ratio *E*/*H* (*H* = hydrostatic strain, *E* = equivalent strain), as expressed in Equation (1). Additionally, macroscopic stress state information is obtained from the reaction force of the moving rigid boundary and is expressed as a stress measurement, as in Equation (2).

(1) $$H=\left(E_{z}+E_{x}\right)\;\mathrm{and}\;H=\frac{1}{2}\left(E_{z}-E_{x}\right)$$

In Equation (1), *H* is the hydrostatic strain, and *E* the equivalent strain in the 2D plane.

(2) $$\sum_{m}=\frac{1}{2}(\sum_{z}+\sum_{x})\;\mathrm{and}\;\sum =(\sum_{z}-\sum_{x})$$

In Equation (2), *∑_m_* is the hydrostatic component, and *∑* is the deviatoric stress response.

### 2.3. Powder Compression Molding Analysis

The compressibility of the powder varies depending on the variables of powder compression molding, and the representative results for confirming compressibility include the forming load, relative density, effective stress, and average stress. Forming load refers to the repulsive force in the compression direction of the continuum, and as the forming load increases, compressibility decreases. Relative density indicates the effectiveness of the powder compression. In general, as the relative density increases, the porosity between powder particles decreases, and the strength of the material increases. In addition, the greater the deviation in relative density, the more uneven the density of the final molded body, leading to defects such as cracks. Equation (3) expresses effective stress by using the Von Mises yield theory, that is, the average vertical stress per unit area. Molded products can be predicted to fail when the analytically obtained effective stress is greater than the yield stress of the material, as well as if there exist areas in which the deviation changes rapidly. Equation (4) expresses the average stress, which does not include the shear stress that occurs inside a solid under constant conditions. The negative and positive directions represent compressive and tensile stresses, respectively.

(3) $$\sigma_{\mathrm{VM}}=\sqrt{\frac{1}{2}\left[{\left(\sigma_{\mathrm{xx}}-\sigma_{\mathrm{yy}}\right)}^{2}+{\left(\sigma_{\mathrm{yy}}-\sigma_{\mathrm{zz}}\right)}^{2}+{\left(\sigma_{\mathrm{zz}}-\sigma_{\mathrm{xx}}\right)}^{2}+3\left(\tau_{\mathrm{xy}}^{2}+\tau_{\mathrm{yz}}^{2}+\tau_{\mathrm{xz}}^{2}\right)\right]}$$

(4) $$\sigma_{h}=\frac{\sigma_{\mathrm{xx}}+\sigma_{\mathrm{yy}}+\sigma_{\mathrm{zz}}}{3}$$

In the equations above, $\sigma_{\mathrm{VM}}$ is the Von Mises stress; $\sigma_{h}$ the mean stress; $\sigma_{\mathrm{xx}}$, $\sigma_{yy}$, and $\sigma_{\mathrm{zz}}$ the uniaxial stresses along the respective axes; and $\tau_{\mathrm{xx}}$, $\tau_{yy}$, and $\tau_{\mathrm{zz}}$ the shear stresses along the respective axes.

### 2.4. Flow Chart of the Study

**Figure 1:**
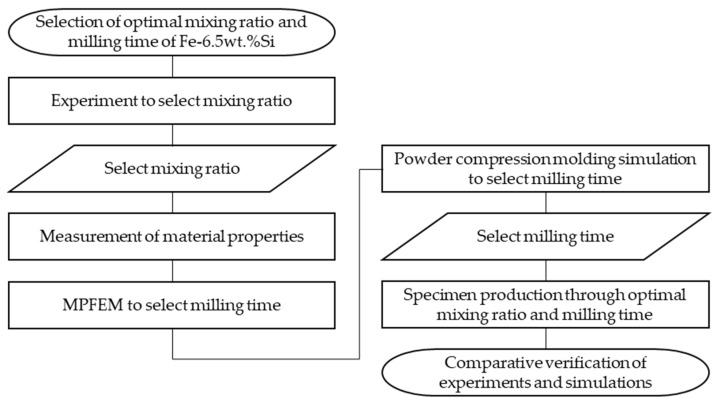
Flow chart of the study.

A flow chart of the study is depicted in Figure 1.

## 3. Material Properties of Fe-6.5wt.%Si

Because measuring the mixing ratio and physical properties of Fe-6.5wt.%Si powder according to its milling time is time-consuming and expensive, we selected the mixing ratio by conducting experiments.

### 3.1. Experiment to Select Mixing Ratio

In this study, we attempted to experimentally evaluate the compressibility in terms of the ratio of hybrid (water + gas) atomizing powder and gas atomizing powder by using the process conditions of high-temperature compression molding proposed in a previous study: molding temperature of 500 °C and molding speed of 1 mm/s [26]. Accordingly, as listed in Table 1, four ratios of each powder were selected from 100:0 to 30:70. In addition, the quantity of charge was set to 8 g to achieve the target density of 7.2 g/cm^3^.

#### Results

**Figure 2:**
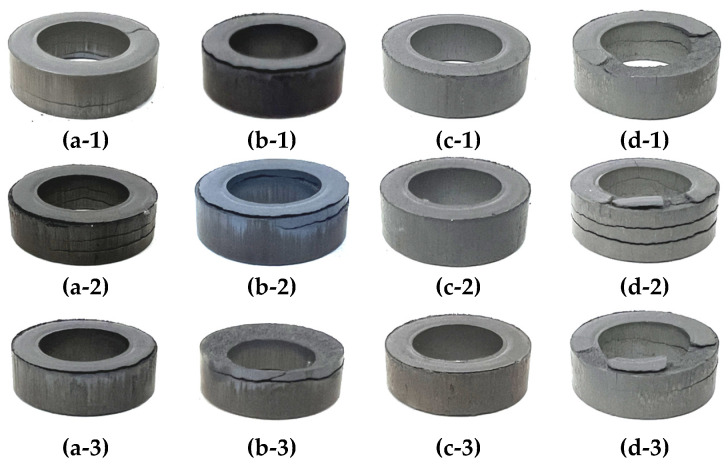
Appearance of toroidal cores: (**a-1**–**a-3**) Case 1—100:0; (**b-1**–**b-3**) Case 2—70:30; (**c-1**–**c-3**) Case 3—50:50; (**d-1**–**d-3**) Case 4—30:70.

To evaluate the compressibility of the toroidal core in terms of the mixing ratio, three products each were molded using the powders mixed in each of the four aforementioned ratios, as depicted in Figure 2. The appearance and density of the final molded products were checked.

Based on the results, it was confirmed that cracks occurred in all products, except those prepared with the powder with the 50:50 mixing ratio of hybrid atomizing powder and gas atomizing powder. In particular, the product prepared using the 30:70 ratio had the most cracks.

Because the density of the molded products with cracks could not be measured, the product prepared using the 50:50 powder, which did not have cracks, was coated with paraffin, and its density was measured using an underwater method. The respective densities are 7.036 g/cm^3^, 7.031 g/cm^3^, and 7.013 g/cm^3^, and the standard deviation is 0.012 g/cm^3^.

Accordingly, the 50:50 powder mixing ratio was used to determine compressibility in terms of milling time.

**Table 1:** Powder mixing ratio for toroidal core molding.

Case No.	Composition Ratio	
	Hybrid Atomizing Fe-6.5wt.%Si (%)	Gas Atomizing Fe-6.5wt.%Si (%)
1	100	0
2	70	30
3	50	50
4	30	70

### 3.2. Mechanical Properties

#### High-Temperature Compression Test and Strain–Stress Curve

**Figure 3:**
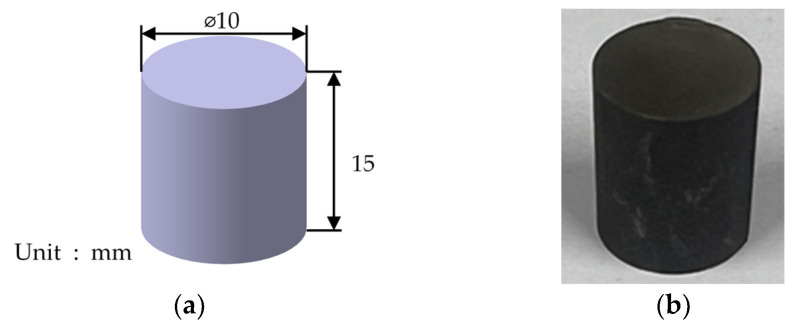
Specimen geometry: (**a**) model of specimen used in high-temperature compression test; (**b**) specimen used in high-temperature compression test.

**Figure 4:**
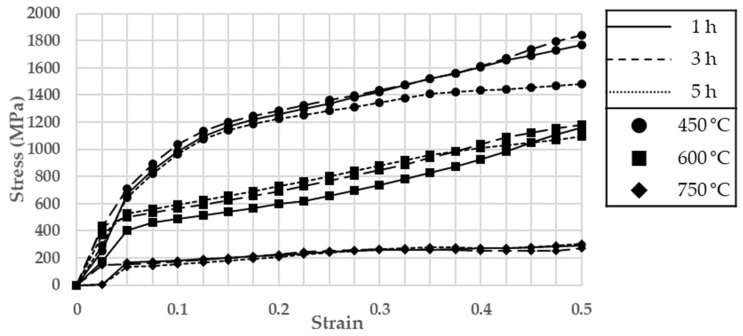
S-S curve of gas atomizing Fe-6.5wt%Si.

A high-temperature compression test was performed by following the ASTM E 209 test standard [33] and using a dynamic thermal mechanical testing machine (Gleeble 3800-GTC, Gleeble, Poestenkill, NY, USA), and its specifications are summarized in Table A1. Moreover, cylindrical specimens were used in the test, and their geometry is depicted in Figure 3. The measurement temperature was around 500 °C, at which Fe-6.5wt.%Si is known to have the best formability. Therefore, temperatures of 450 °C, 600 °C, and 750 °C were selected, and the powder ratio selected through toroidal core molding was used [2]. The compression speed was 0.1 mm/s. Using the high-temperature compression test results, an S-S curve was plotted for milling times of 1 h, 3 h, and 5 h and is depicted in Figure 4.

According to the results of the high-temperature compression test, Fe-6.5wt.%Si exhibited brittleness, which can largely be attributed to the brittleness of Si. Moreover, for all milling times, stress decreased as temperature increased, and the lowest stress was achieved when the gas atomizing powder was milled for 3 h.

### 3.3. Thermal Properties

#### 3.3.1. Thermal Diffusivity and Thermal Conductivity

**Figure 5:**
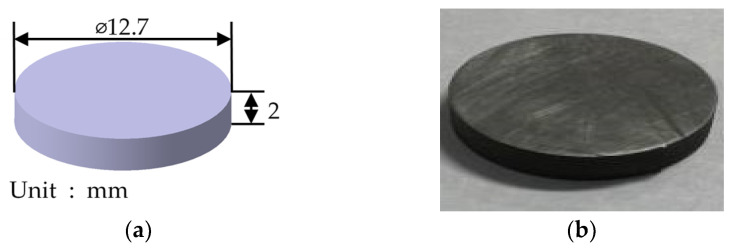
Specimen geometry: (**a**) model of thermal conductivity specimen; (**b**) thermal conductivity specimen.

A thermal conductivity test was performed by following the ASTM E 1461 test standard [34], and the test device was an LFA427 (NETZSCH, Selb, Bayern, Germany), and its specifications are summarized in Table A2. The measurements were performed from 25 °C to 900 °C. A disk-type specimen was used, and its dimensions are shown in Figure 5. The results of the thermal diffusivity and thermal conductivity tests are depicted and summarized in Table 2.

Thermal conductivity increased as the test temperature increased, and in particular, it deviated the most when the temperature increased from 500 °C to 600 °C. It is predicted that during high-temperature compression molding, as the molding temperature increases, the molding load decreases and molding improves. However, as the molding temperature increases, the cycle time (CT) increases, and the required energy increases. Therefore, it is expected that an appropriate molding temperature will be required.

#### 3.3.2. Thermal Expansion Coefficient

**Figure 6:**
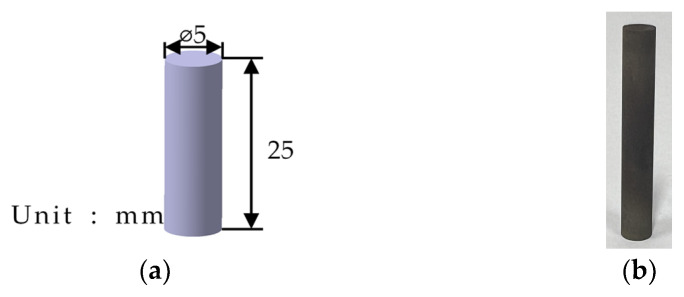
Specimen geometry: (**a**) model of thermal expansion coefficient test specimen; (**b**) thermal expansion coefficient test specimen.

A thermal expansion coefficient test of Fe-6.5wt.%Si was performed by following the ASTM E 228 test standard [35]. The test device used was the DIL402HT (NETZSCH, Selb, Bayern, Germany); its specifications are summarized in Table A3 and its specimen dimensions are shown in Figure 6. The measurements were performed at temperatures of 100 °C–900 °C. The test results are summarized in Table 3.

According to the results, the thermal expansion coefficient of Fe-6.5wt.%Si increased as the test temperature increased. Therefore, the degree of deformation of molded products may increase as the molding temperature increases during high-temperature compression molding.

### 3.4. Particle Size Analysis

**Figure 7:**
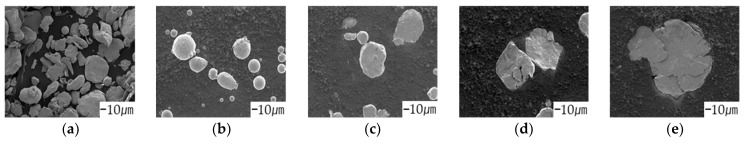
SEM of powders: (**a**) hybrid atomizing—0 h; (**b**) gas atomizing—0 h; (**c**) gas atomizing—1 h; (**d**) gas atomizing—3 h; (**e**) gas atomizing—5 h.

In this work, milling was performed to change the particle size of spherical Fe-6.5wt.%Si gas atomizing particles. An Attr-3C (Attrition mill, DAEWHA TECH, Yongin-si, Gyeonggi-do, Republic of Korea) was used as the test device, and its specifications are summarized in Table A4. The milling speed was set to 600 RPM, and the milling times were 1 h, 3 h, and 5 h. Scanning electron microscopy (SEM, JSM-7900F, JEOL, Akishima, Tokyo, Japan) was used to verify the results of the milling test, and its specifications are summarized in Table A5. In addition, particle size analysis was performed to confirm the particle sizes of the hybrid atomizing Fe-6.5wt.%Si and gas atomizing Fe-6.5wt%Si as a function of milling time. An LS 13 320 (Beckman Coulter, Brea, CA, USA) particle size analyzer was used for this purpose, and its specifications are summarized in Table A6. The results, summarized in Table 4, indicate that the particle size increased as the milling time increased. Moreover, the SEM analysis results are depicted in Figure 7.

The results of the particle size analysis of the gas atomizing Fe-6.5wt.%Si as a function of milling time confirm that the average particle size increased as the milling time increased. This is a phenomenon that occurs when powder undergoes repeated plastic deformation and agglomerates.

## 4. Simulations

### 4.1. MPFEM

#### 4.1.1. Analysis Conditions

**Figure 8:**
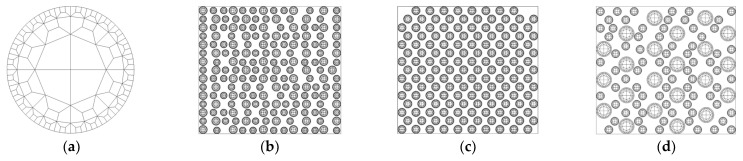
MPFEM model: (**a**) mesh; (**b**) milling time of 1 h; (**c**) milling time of 3 h; (**d**) milling time of 5 h.

In this analysis, a mesh consisting of 132 elements and 169 nodes, developed by Procopio and Zavaliangos, was used, as illustrated in Figure 8a. Compression molding of an actual powder occurs in three dimensions, but calculations for modeling such a process would be time consuming. Therefore, we proceeded with the analysis in two dimensions [31,32].

Using the 50:50 mixing ratio of hybrid atomizing powder to gas atomizing powder, as selected in Section 3, the diameter of the hybrid atomizing powder particles was assumed to be 1 mm based on the particle size analysis results obtained in Section 3. Moreover, the particle sizes of the gas atomizing powders milled for 1 h, 3 h, and 5 h were assumed to be 0.814 mm, 1.012 mm, and 1.830 mm, respectively.

To analyze compressibility in terms of milling time, the ratio (*A_TP_*/*A_C_*) of total particle area (*A_TP_*) to container area (*A_C_* = 247.5 mm^2^) was defined. The *A_TP_*/*A_C_* values of the gas atomizing powders milled for 1 h, 3 h, and 5 h were 0.4702, 0.4756, and 0.4761, respectively. This difference in area ratio was attributed to the need for regular particle arrangement within the container.

Therefore, the number of particles was 75 for all the hybrid atomizing powders, while the numbers of particles in the gas atomizing powders milled for 1 h, 3 h, and 5 h were 113, 73, and 22, respectively. The container size was 16.5 × 15 mm, and a one-way compression process identical to the experiment was considered. The analysis model is depicted in Figure 8.

Regarding the molding conditions, the process conditions for high-temperature compression molding proposed in a previous study were used, namely a molding temperature of 500 °C and molding speed of 1 mm/s, and the friction coefficient between each particle was set to 0.056 in all analyses. Compression molding was performed using ABAQUS Explicit.

#### 4.1.2. Results of MPFEM

**Figure 9:**
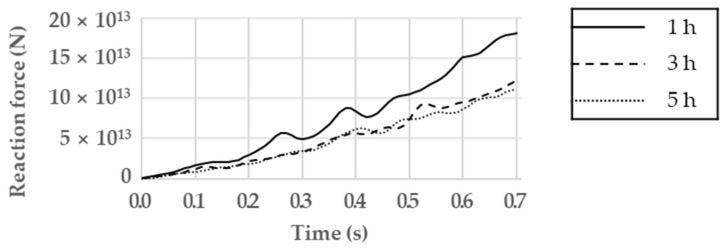
Reaction force as a function of milling time.

Figure 9 presents a comparison of reaction forces as a function of the milling time of the gas atomizing powder. According to the analysis results, the reaction force increased as the compression time increased. In addition, as the milling time increased, the molding load decreased, and it was confirmed that the difference between the reaction forces of the gas atomizing powders milled for 3 h and 5 h was small.

**Figure 10:**
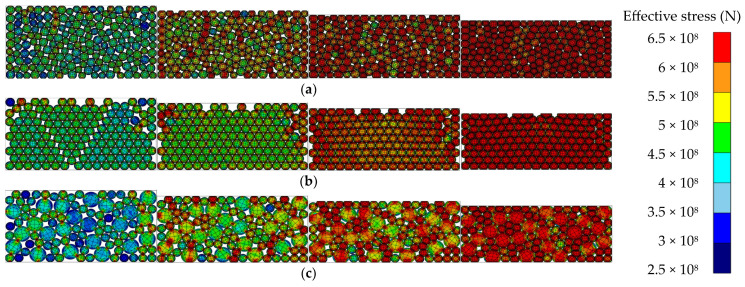
Effective stress as a function of milling time: (**a**) milling time of 1 h; (**b**) milling time of 3 h; (**c**) milling time of 5 h.

**Figure 11:**
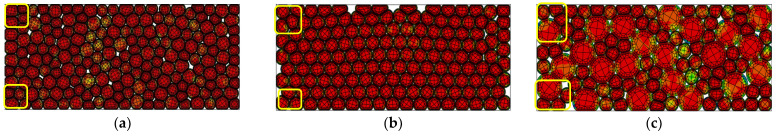
Compaction load as a function milling time: (**a**) milling time of 1 h; (**b**) milling time of 3 h; (**c**) milling time of 5 h.

Figure 10 shows the effective stress of all the powder particles over time. As time passed, the effective stress of the entire system increased, and the effective stress appeared large at relatively small particles and the die wall. Figure 11 presents the local effective stress, as well as the area in which friction occurs with the die wall and the area surrounding the large particles; the maximum local effective stress values are listed in Table 5. The results confirm that as the milling time of the gas atomizing powder increased, the repulsive force applied to the upper punch decreased.

Based on these results, the gas atomizing powder milled for 5 h had the best compressibility. However, in view of energy efficiency, the powder milled for 3 h, which yielded similar results, was selected.

### 4.2. Powder Compression Molding Analysis

#### 4.2.1. Analysis Conditions

In this analysis, the process conditions for high-temperature compression molding proposed in previous studies were used (molding temperature of 500 °C and molding speed of 1 mm/s), and the MPFEM was performed under the same conditions. The material property results presented in Section 3 were used.

**Figure 12:**
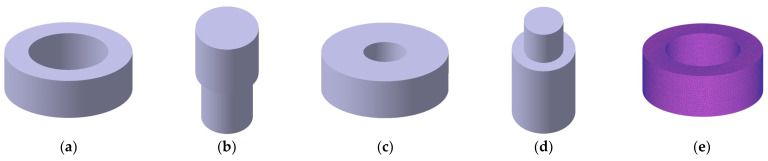
FEM models of toroidal core: (**a**) toroidal core; (**b**) upper punch; (**c**) die; (**d**) lower punch; (**e**) mesh.

Figure 12 presents the results of the 3D modeling of high-temperature compression molding, which was performed using CATIA V5 to check the compressibility of the toroidal core, upper punch, lower punch, die, and workpiece. The weight of the modeled workpiece was 8 g, which was equal to that of the experiment; the dimensions of the workpiece are listed in Table 6.

DEFORM-3D was used for high-temperature compression molding analysis. In this analysis, the object type of the workpiece was porous; the initial density was set to 4.5 g/cm^3^, which was the apparent density; and the relative density was set to 0.6. Moreover, in total, 163,295 tetrahedral mesh elements were used. The mesh shape is depicted in Figure 12e.

#### 4.2.2. Results of Powder Compression Molding Analysis

**Figure 13:**
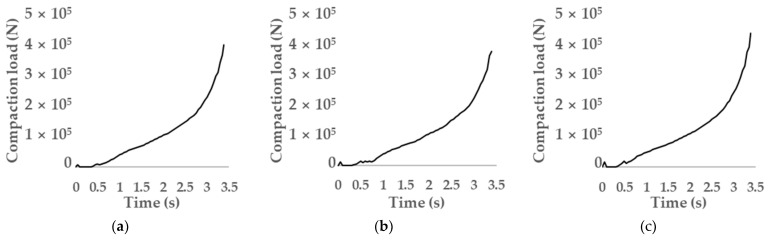
Compaction load as a function of milling time: (**a**) milling time of 1 h; (**b**) milling time of 3 h; (**c**) milling time of 5 h.

**Figure 14:**
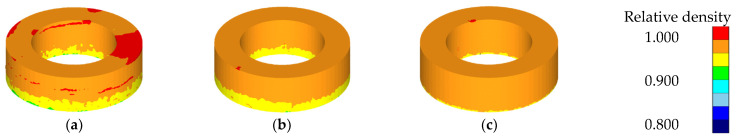
Relative density as a function of milling time: (**a**) milling time of 1 h; (**b**) milling time of 3 h; (**c**) milling time of 5 h.

**Figure 15:**
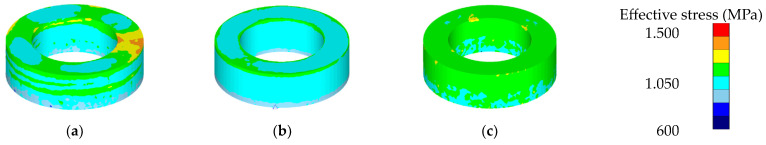
Effective stress as a function of milling time: (**a**) milling time of 1 h; (**b**) milling time of 3 h; (**c**) milling time of 5 h.

**Figure 16:**
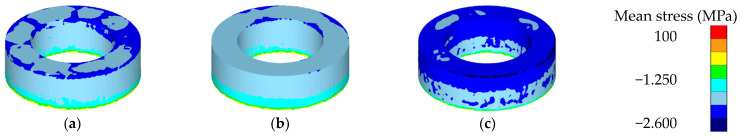
Mean stress as a function of milling time: (**a**) milling time of 1 h; (**b**) milling time of 3 h; (**c**) milling time of 5 h.

Figure 13, Figure 14, Figure 15 and Figure 16 depict compaction load, relative density, effective stress, and mean stress as a function of the gas atomizing powder, respectively. Furthermore, the analysis results are summarized in Table 7. In terms of average relative density and standard deviation as network characteristics, the relative density, effective stress, and difference between the maximum and minimum values of mean stress on the powder milled for 3 h had the best compressibility.

Compaction load refers to the force needed for the compression molding of powder. In general, the higher the forming load, the lower the energy efficiency. The maximum compaction load of the powder milled for 3 h was the lowest, followed by those of the powders milled for 1 h and 5 h, in that order.

The relative densities of the powders milled for 3 h and 5 h were the same at 0.96, and the difference between the maximum and minimum relative densities of the elements was the same. The standard deviation of the powder milled for 5 h was the lowest.

The average effective stress of the powder milled for 3 h was the lowest, followed by those of the powders milled for 1 h and 5 h, in that order. The maximum effective stress and minimum relative density of the elements of the powder milled for 3 h were the lowest, followed by those of the powders milled for 5 h and 1 h, in that order. The standard deviation of the powder milled for 3 h was the lowest.

The average mean stress of the powder milled for 3 h was the lowest, followed by those of the powders milled for 1 h and 5 h, in that order. The maximum and minimum mean stresses of elements of the powder milled for 3 h were the lowest, followed by those of the powders milled for 5 h and 1 h, in that order. The standard deviation of the powder milled for 3 h was the lowest.

This indicates that the closer the particle size ratio of powders with different particle sizes is to 1, the better the compressibility of the powder.

## 5. Experimental Verification of Analysis Results

The results of the MPFEM and compression molding analysis were compared to the results of the experiment under the same conditions, that is, an optimal mixing ratio of 50:50 and an optimal milling time of 3 h, for verification.

### 5.1. Density

**Figure 17:**
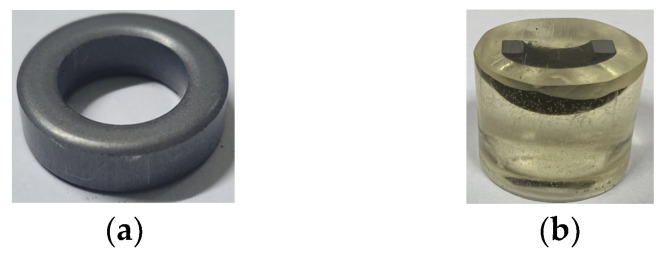
Specimens: (**a**) Density specimen; (**b**) SEM specimen.

The toroidal core was coated with paraffin and tested by following the KS-D-0033 test standard [36], one of the underwater methods. The test was performed using an XS204 (METTLER-TOLEDO, Columbus, OH, USA), and the specifications of this device are summarized in Table A7. The dense specimen is depicted in Figure 17a. The respective densities are 7.178 g/cm^3^, 7.195 g/cm^3^, and 7.183 g/cm^3^, and the average density is 7.185 g/cm^3^, which is close to the average density result of the compression molding analysis, that is, 7.2 g/cm^3^, with an error rate of 0.2%.

### 5.2. SEM Analysis

**Figure 18:**
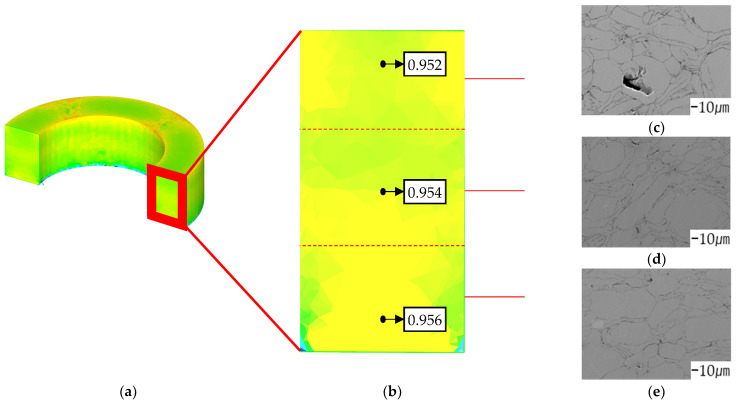
Internal relative density: (**a**) internal shape of toroidal core; (**b**) relative density of toroidal core; (**c**) SEM of upper point; (**d**) SEM of middle point; (**e**) SEM of lower point.

The SEM specimen was produced as shown in Figure 17b. As depicted in Figure 18, points located at 5 mm, 3 mm, and 1 mm from the bottom of the molded product were defined as the top, middle, and bottom, and SEM analysis was performed. According to the results of the compression molding analysis, internal density was the highest at the bottom, followed by those at the middle and top, in that order. The results of the compression molding analysis and SEM analysis exhibited consistent trends.

## 6. Discussion and Conclusions

In this study, the compressibility of powder was evaluated as a function of the mixing ratio of Fe-6.5wt%Si and milling time.

1. The mixing ratio of Fe-6.5wt.%Si hybrid atomizing powder to Fe-6.5wt.%Si gas atomizing powder was selected through high-temperature compression molding of the toroidal core. According to the result, the mixing ratio of 50:50 provided the best compressibility.
2. In the FEM analysis performed based on the selected mixing ratio of 50:50, the physical properties of the gas atomizing powder were measured and presented as a function of milling time (1 h, 3 h, and 5 h).
3. By using the MPFEM, the compressibility of the gas atomizing powder was analyzed as a function of milling time, and the difference in reaction force between the powders milled for 3 h and 5 h was found to be extremely small. Therefore, the powder milled for 3 h was judged to be superior in terms of energy efficiency.
4. By performing a high-temperature compression molding analysis, the compressibility of the gas atomizing powder was analyzed as a function of milling time, and the powder milled for 3 h was judged to be the best.
5. Following the MPFEM and high-temperature compression molding analysis, the excellent compressibility of the gas atomizing powder milled for 3 h was verified through high-temperature powder compression molding, and it was confirmed that the internal density trend was the same using density and SEM.

The compressibility of gas atomizing powder was analyzed using the MPFEM and high-temperature compression molding, with reaction force, compaction load, density, effective stress, and mean stress evaluated as a function of milling time. Powders milled for 3 h exhibited optimal characteristics in terms of reaction force and compaction load, making them energy-efficient and indicating superior compressibility. Consequently, a mix of hybrid atomizing and gas atomizing powders, each at 50% and milled for 3 h, was chosen for further testing.

The selected powder mix was used in toroidal core high-temperature compression molding, resulting in a product without shape defects and with a density of 7.185 g/cm^3^. Validation experiments, including density measurements by scanning electron microscopy (SEM) and internal density trend analysis, confirmed the compressibility of the selected powder mixtures. The experimental density closely matched the simulated density of 7.2 g/cm^3^ with a minimal error rate of 0.2%, and density trends from the top to the bottom of the toroidal core were consistent between the simulations and experimental results. This analysis demonstrated the selected powder’s superior compressibility.

Before the developed powder can be employed in mass production, it must be verified further by conducting tests under various process conditions.

## Figures and Tables

**Table 2:** Thermal conductivity test results as a function of temperature.

Temperature (°C)	Thermal Diffusivity (mm^2^/s)	Specific Heat (J/gK)	Thermal Conductivity (W/mK)
25	4.201	0.452	14.2
100	4.340	0.540	17.6
200	4.497	0.565	19.0
300	4.592	0.580	20.0
400	4.601	0.597	20.6
500	4.514	0.639	21.6
600	4.212	0.907	28.6
700	3.763	1.070	30.2
800	4.173	0.973	30.4
900	4.512	0.926	31.3

**Table 3:** Thermal expansion coefficient test results as a function of temperature.

Temperature (°C)	Coefficient of Thermal Expansion (1/°C)
100	1.127
200	1.190
300	1.260
400	1.307
500	1.354
600	1.418
700	1.468
800	1.503
900	1.532

**Table 4:** Results of particle size analysis.

Description		Particle Size (@D50) (µm)
Atomizing	Milling Time (h)	
Hybrid	0	56.58
Gas	0	23.77
	1	46.16
	3	57.36
	5	103.70

**Table 5:** Maximum local effective stress determined through MPFEM.

Time (s)	Maximum Local Effective Stress (N)		
	1 h	3 h	5 h
0.7	6.02 × 10^8^	5.90 × 10^8^	5.69 × 10^8^

**Table 6:** Specifications of toroidal core model.

Description	Value (mm)
Outer diameter	20.300
Inner diameter	12.700
Height	9.025

**Table 7:** Results of compaction simulation.

Description		Value		
		1 h	3 h	5 h
Compaction load (N)	Maximum	398,778	377,069	436,395
Relative density	Average	0.96	0.96	0.96
	Maximum	0.96	0.98	0.99
	Minimum	0.88	0.89	0.90
	Standard deviation	0.01	0.01	0.00
Effective stress (MPa)	Average	1027.53	994.24	1086.72
	Maximum	1375.01	1133.88	1330.27
	Minimum	743.57	806.23	890.98
	Standard deviation	56.58	32.19	39.04
Mean stress (MPa)	Average	−1711.27	−1614.60	−1933.67
	Maximum	−359.26	−326.59	−484.65
	Minimum	−2316.33	−2236.49	−2410.46
	Standard deviation	217.81	194.94	214.47

## Data Availability

Data are contained within the article.

## Appendix A

**Table A1:** Specifications of Gleeble 380-GTC.

Description		Value
Force	Maximum compressive force	20 metric tons
	Maximum tensile force	10 metric tons
Stroke	Maximum stroke	125 mm
	Stroke rate	0.001–2000 mm/s
Temperature control	Maximum temperature	3000 °C
	Maximum heating rate	10,000 °C/s
	Maximum quenching rate	10,000 °C/s

**Table A2:** Specifications of LFA427.

Description		Value
Temperature range	Graphite furnaces	25–2000 °C/2800 °C
Heating rates		0.01–50 K/min
Measurement range	Thermal diffusivity	0.01–1000 mm^2^/s
	Thermal conductivity	0.1–2000 W/(m·K)

**Table A3:** Specifications of DIL402HT.

Description	Value
Temperature range	RT–1.150 °C
Heating rates	0.001 K/min–50 K/min
Measurement range	±500 µm

**Table A4:** Specifications of Attr-3C.

Description	Value
Vessel capacity	3 L (jacket type)
Vessel material	Zr_2_O_3_, SUS, WC
PIN material	Zr_2_O_3_, SUS, WC
Maximum speed	1200 rpm

**Table A5:** Specifications of JSM-7900F.

Description	Value
Electron gun	Schottky field emission
Accelerating voltage	0.2–30 kV
Resolution	0.7 nm (15 kV), 0.8 nm (1 kV), 1.0 nm (0.5 kV)
Magnification	×25–1,000,000

**Table A6:** Specifications of LS 13 320.

Description	Value
Measurement range	0.04–2.000 µm
Light source	Solid-state laser (750 nm)
PIDS lamp	450, 600, 900 nm
Size classification count	116

**Table A7:** Specifications of XS204.

Description	Value
Maximum capacity	220 g
Readability	0.1 mg
Repeatability, typical	0.04 mg
Minimum weight (USP, 0.1%, typical)	82 mg
